# ZNF350 gene polymorphisms promote the response to Peg-IFNα therapy through JAK-STAT signaling pathway in patients with chronic hepatitis B

**DOI:** 10.3389/fimmu.2024.1488055

**Published:** 2024-11-07

**Authors:** Jianhui Guo, Shaoying Guo, Tianbin Chen, Yongbin Zeng, Ya Fu, Qishui Ou

**Affiliations:** ^1^ Department of Laboratory Medicine, Fujian Key Laboratory of Laboratory Medicine, Gene Diagnosis Research Center, Fujian Clinical Research Center for Clinical Immunology Laboratory Test, The First Affiliated Hospital, Fujian Medical University, Fuzhou, China; ^2^ Department of Laboratory Medicine, National Regional Medical Center, Binhai Campus of the First Affiliated Hospital, Fujian Medical University, Fuzhou, China

**Keywords:** hepatitis B virus, single nucleotide polymorphism, zinc finger protein 350, Peg-IFNα, therapeutic effect

## Abstract

**Background:**

The varying individual responses to Pegylated interferon-α (Peg-IFNα) in patients with chronic hepatitis B (CHB) pose significant hurdles in treatment optimization, and the underlying mechanisms remain unclear.

**Objective:**

We aimed to identify genetic polymorphisms influencing the efficacy of Peg-IFNα in patients with HBeAg-positive CHB, with the goal to predict Peg-IFNα response before treatment.

**Methods:**

We employed an Asian Screening Array analysis involving 124 HBeAg-positive CHB patients treated with Peg-IFNα. We conducted assessment of immunological markers and mRNA expression of pivotal genes, establishing correlations with SNPs, functional genes of SNPs, and efficacy of Peg-IFNα therapy. *In vitro* experiments were performed to verify the functional involvement of the candidate SNPs.

**Results:**

The G allele presented in rs2278420 and rs6509607 were significantly more common in patients who achieved a complete response (CR) compared to those who had a suboptimal response (SR), and linked to an increased rates of HBeAg loss following Peg-IFNα treatment (all *p* < 0.05). Additionally, the mRNA level of ZNF350 varied notably across different genotypes of both SNPs as determined by eQTL analysis, and showed higher expression in patients achieved a SR (all *p* < 0.05). *In vitro* investigations with IFNα stimulation showed that the mRNA level of SOCS3 was elevated in patients with rs2278420 genotype AA, similarly, mRNA levels of PKR, STAT2, SOCS1, SOCS3, PIAS1, PTPN6 and TRIM8 were heightened in patients with rs6509607 genotype AA compared to those with genotypes (AG+GG) (all *p* < 0.05).

**Conclusion:**

The G allele of rs2278420 and rs6509607 were associated with mRNA level of ZNF350, with an increased probability of Peg-IFNα response in HBeAg-positive CHB patients, likely through the modulation of JAK-STAT signaling pathway.

## 1 Introduction

Chronic hepatitis B virus (HBV) infection remains a significant global health concern, affecting approximately 296 million individuals worldwide (1). Pegylated interferon-α (Peg-IFNα) and nucleos(t)ide analogues (NAs) are currently the mainstream therapies for chronic hepatitis B (CHB) (2). Despite their availability, only a minority of patients achieve the sustained virological response, and the underlying reasons for this variability in treatment outcomes remain largely unclear (3–5).

Recent studies have begun to uncover the role of single nucleotide polymorphisms (SNPs) in host genes in influencing both susceptibility to HBV infection and the individual differences in interferon response. For example, certain genotypes such as AT of rs77076061 and GG of rs1979262 have been identified as protective factors to HBsAg-positive patients, likely through influencing C19orf66 expression by alteration of transcription factors binding (6). The genotype CC of rs10838543 in the TRIM22 gene was associated with a beneficial response to PegIFN-α through the modulation of immunoinducible cytokines (7). The rs7519753 allele C was reported to be correlated to increased expression of the TP53BP2 protein, and associated with a higher probability of serum HBsAg loss in CHB patients following Peg-IFNα treatment (8). However, the genetic determinants that contribute to the therapeutic outcomes with Peg-IFNα and their underlying mechanisms remain partially understood, warranting further exploration.

Genome-wide association studies (GWAS), widely used to investigate complex traits genetics, disease biology, and new therapeutic development (9, 10), have recently incorporated an Asian Screening Array (ASA) program. This program includes East Asia’s largest whole-genome reference dataset, has newly identified over 120,000 clinically significant variants as well as drug efficacy-related variants, HLA regional variants, and GWAS susceptibility variants.

In this study, we conducted an ASA analysis on HBeAg-positive CHB patients who treated with Peg-IFNα, and identified two new loci (rs2278420 and rs6509607) associated with HBeAg loss on chromosome 19. Furthermore, these two SNPs were found to be associated with the regulation of IFNα signaling pathway *in vitro.* Additionally, through expression quantitative trait locus (eQTL) analysis and mRNA detection, we confirmed that the differential expression of ZNF350, a gene where rs2278420 is located on and 1.5 kb downstream of rs6509607, across different genotypes of SNPs and was associated with the varying efficacy of Peg-IFNα treatment. Our findings highlighted two new genetic biomarkers associated with the efficacy of PegIFN-α therapy, providing new ideas in guiding therapy selection for CHB patients.

## 2 Materials and methods

### 2.1 Subjects

A total of 124 HBeAg-positive CHB patients treated with Peg-IFNα from the First Affiliated Hospital of Fujian Medical University were enrolled in this study. The inclusion criteria were as follows: i) a previous lack of any antiviral therapy; ii) age ≥ 16 years old; iii) HBsAg positive for more than 6 months; iv) HBeAg positive; v) HBV DNA ≥ 2000 IU/mL at baseline; vi) serum alanine aminotransferase (ALT) ≥ 40U/L; vii) patients were treated with Peg-IFNα for at least 48 weeks and subsequently observed until 72 weeks. Patients with the other infectious liver diseases or autoimmune diseases were excluded. Of these 124 patients, 63 patients were treated with Peg-IFNα monotherapy while the other 61 patients were received combination therapy of Peg-IFNα and NAs. All subjects were divided into a complete response (CR) group and a suboptimal response (SR) group. CR was simultaneously satisfied with HBV DNA persistently < 500 IU/mL, HBeAg loss, and ALT < 40 U/L, while patients who failed to meet either one of the criteria above were considered SR. For the cytological experiments, we enrolled 81 patients who were newly diagnosed with HBV infection and tested positive for HBeAg in physical examination center of the First Affiliated Hospital of Fujian Medical University. This study was approved by the Ethics Committee of the First Affiliated Hospital of Fujian Medical University.

### 2.2 Genotyping

Genomic DNA was extracted from 2mL of EDTA-anticoagulated peripheral blood using standard procedures of QIAamp DNA Mini Kit (QIAGEN, Germany). Genotyping of a total of 124 CHB patients were performed using high-throughput Infinium Asian Screening Array-24 (ASA) v1.0 Kit (Illumina, Inc., San Diego, CA, USA) and were detected by BioMiao Biological Technology (Beijing) Co., Ltd. All of the raw data was read by Illumina Genomstudio software. Sanger sequencing by BGI (Shanghai) Technology Co., Ltd was performed to identify the target SNPs of 81 HBV infected patients, and primers were designed by Oligo 7 software (**Supplementary Table S1**).

### 2.3 Measurement of serum HBV DNA, HBsAg, HBeAg and ALT

Serum HBsAg and HBeAg were quantified using the automated chemiluminescent microparticle immunology analyzer (Abott Architect-I4000, Chicago, IL, USA). Biochemical indicators such as Serum ALT and AST were detected by automatic biochemical analyzer ADVIA 2400 (Siemens, Munich, Germany). HBV DNA was detected with Roche LightCyCler480 (Roche Corporation, Basel, Switzerland) and quantitative real-time PCR method (Sansure Biotech Inc., Hunan, China).

### 2.4 eQTL analysis

To identify the target genes associated with SNPs, we performed the eQTL analysis through the GTEx database (https://gtexportal.org/), a project collected samples from non-diseased tissue sites. The correlations between SNPs and ZNF350 expression in the whole blood and other tissue of GTEx were analyzed through the “GTEx eQTL Calculator”.

### 2.5 Cell culture and stimulation

The EDTA-anticoagulated peripheral blood samples were collected from 81 patients who diagnosed with HBV infection firstly. Freshly isolated PBMCs were cultured on 96-well plates at 37 °C in a 5% CO_2_ incubator for 18 hours and subsequently incubated with IFNα (1,000 U/mL; Beyotime Biotechnology) for 6 hours. All cells were collected for RNA extraction using TRIzol Reagent (Thermo Fisher Scientific, USA).

### 2.6 Quantitative reverse transcription-polymerase chain reaction

RNA reverse transcription was performed with TransStart® Green qPCR Super Mix (TransGen Biotech, Beijing, China). The qPCR was performed with 2 plus SYBR Green PCR mix (Takara, Tokyo, Japan) on the ABI Step One Plus RT-PCR system (Applied Biosystems, CA, USA) with the program as follows: 30 s at 95 °C, then 40 cycles of 5 s at 95 °C and 30 s at 60 °C. Primers were designed by Oligo 7 software (**Supplementary Table S1**). The data of qRT-PCR were analyzed by the 2^-ΔCt^ method.

### 2.7 Statistical analysis

Clinical indicators were represented as mean ± SD or indicated otherwise. The repeated measurement data were analyzed by generalized estimating equation. Student’s T test or Mann-Whitney U test was used for comparing two independent groups, and chi-square (χ^2^) test was used for categorical data. The correlation analysis was performed by the Pearson analysis. Data were analyzed by SPSS20.0 software (SPSS, Inc.) and figures were made by GraphPad Prism 6.0 (GraphPad Software, Inc.). *P* < 0.05 was considered statistically significant.

## 3 Results

### 3.1 Cohort characteristics and ASA association analysis

**Figure 1:**
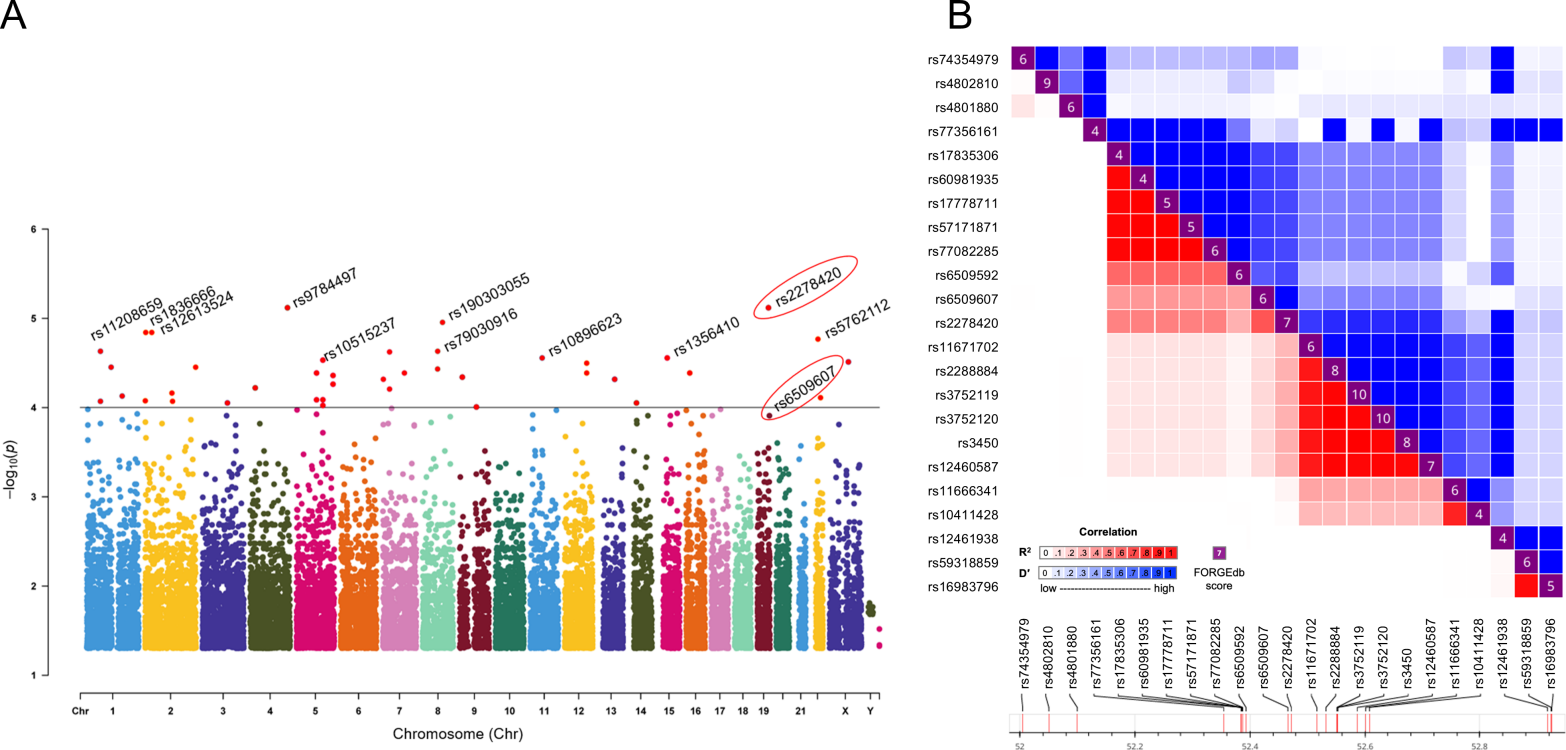
Rs2278420 and rs6509607 were associated with Peg-IFNα treatment efficacy. **(A)** The Manhattan plot showed the location of all significant SNPs in the human genome (UCSC hg37) and *p*-values for ASA analysis. The dots with *p* < 10^-4^ were all marked in red. **(B)** Linkage disequilibrium analysis showed D' and R^2^ for rs2278420 with other SNPs within 500 kb upstream and downstream of rs2278420.

Previous studies demonstrated that baseline levels of HBsAg, HBeAg, HBV DNA, ALT and AST were associated with HBeAg or HBsAg loss in CHB patients following Peg-IFNα treatment (11, 12). However, in our study, there were no significant differences observed in the distribution of gender, age, and baseline levels of serological markers including HBsAg, HBeAg, HBV DNA, ALT, and AST between CR and SR groups (*p* > 0.05). General characteristics of the subjects were shown in **Table 1**. A total of 23,506 SNPs were found to be associated with the efficacy of Peg-IFNα in HBeAg-positive CHB patients (*p* < 0.05, **Figure 1A**). The top 10 SNPs and corresponding *p*-values were summarized in **Table 2**.

**Table 1:** Clinical characteristics of the study cohort.

Baseline Characteristics	CR (n=38)	SR (n=86)	*P*
Male Gender, n (%)	23 (18.5)	63 (50.8)	0.156
Age,y	27.39 ± 4.27	28.05 ± 5.00	0.480
HBsAg (log_10_ IU/mL)	4.10 ± 0.53	4.02 ± 0.61	0.584
HBeAg (log_10_ S/CO)	2.57 ± 0.68	2.77 ± 0.64	0.149
HBV DNA (log_10_ IU/mL)	7.09 ± 1.07	7.27 ± 0.98	0.376
ALT (U/L)	288.86 ± 270.28	274.57 ± 268.86	0.793
AST (U/L)	132.14 ± 99.34	130.60 ± 123.44	0.948
Peg-IFNα monotherapy, n (%)	22 (17.7)	37 (29.8)	
Peg-IFNα add-on NAs, n (%)	15 (12.1)	50 (40.3)	–

Data were expressed as mean ± SD. Statistical significance (*p* < 0.05). ALT, alanine aminotransferase; AST, aspartate aminotransferase; HBsAg, hepatitis B surface antigen; HBeAg, hepatitis B e antigen; Peg-IFNα, pegylated interferon-alfa; NAs, nucleos(t)ide analogues; CR, complete response; SR, suboptimal response.

**Table 2:** Top 10 SNPs associated with Peg-IFNα response.

SNP	Chr	Nearest gene	Allele	Odds ratio	*p*
rs10515237	5	PCSK1	A/G	4.129	4.21×10^-6^
rs2278420	19	ZNF350, ZNF350-AS1	A/G	4.788	7.61×10^-6^
rs9784497	4	NEIL3	T/A	4.788	7.61×10^-6^
rs190303055	8	CDH17	C/T	NA	1.11×10^-5^
rs12613524	2	EHD3	C/T	9.034	1.44×10^-5^
rs1836666	2	LINC01250	C/T	9.034	1.44×10^-5^
rs5762112	22	ENSG00000280445	A/G	3.497	1.71×10^-5^
rs11208659	1	LEPR	A/T	23.86	2.34×10^-5^
rs1571151	1	LEPR	A/C	23.86	2.34×10^-5^
rs11208660	1	LEPR	C/T	23.86	2.34×10^-5^

### 3.2 rs2278420 and rs6509607 are significant loci associated with Peg-IFNα efficacy

The rs2278420, a missense variant, located on 5’-UTR of zinc finger protein 350 (ZNF350) and ZNF350 antisense RNA 1 (ZNF350-AS1), was found to be significantly associated with Peg-IFNα efficacy (*p =* 7.61×10^-6^, **Table 2**). We performed linkage disequilibrium (LD) analysis to sites within 500 kb upstream and downstream of rs2278420, the rs6509607 was found to be in LD with rs2278420 (D' = 1.0, R^2^ = 0.672, **Figure 1B**), which also had a relationship with Peg-IFNα efficacy (*p=*3.77×10^-4^, **Table 3**).

**Table 3:** Genotypes and alleles frequency of rs2278420 and rs6509607 in patients with Peg-IFNα treatment.

SNP	Genotype	CR (n = 38)	SR (n = 86)	*p*	OR (95%CI)
rs2278420					
**Allele**	A	51(67.1)	155 (90.1) (64.4)	**< 0.001**	
	G	25(32.9)	17 (9.9)		0.224 (0.112 - 0.447)
**Genotype**	AA	16 (42.1)	70 (81.4)	**7.61×10^-6^**	
	AG	19 (50.0)	15 (17.4)	**< 0.001**	0.180 (0.076 - 0.430)
	GG	3 (7.9)	1 (1.2)	**0.007**	0.076 (0.007 - 0.781)
**HWE *p* value**		0.340	0.847		
**Dominate**	AA vs GG+AG	16 / 22	70 / 16	**< 0.001**	6.016 (2.591 - 13.968)
**Recessive**	GG vs AA+AG	3 / 35	1 / 85	0.051	0.137 (0.014 - 1.365)
rs6509607					
**Allele**	A	38(57.6)	146(84.9)	**< 0.001**	
	G	28(42.4)	26(15.1)		0.242 (0.127 - 0.459)
**Genotype**	AA	14 (36.8)	61 (70.9)	**3.77×10^-4^**	
	AG	20 (52.7)	24 (27.9)	**0.002**	0.275 (0.120 - 0.632)
	GG	4 (10.5)	1 (1.2)	**0.002**	0.057 (0.006 - 0.554)
**HWE *p* value**		0.420	0.417		
**Dominate**	AA vs GG+AG	14 / 24	61 / 25	**<0.001**	4.183 (1.867 - 9.347)
**Recessive**	GG vs AA+AG	4 / 34	1 / 85	**0.015**	0.100 (0.011 - 0.927)

Bold font indicates statistical significance (*p* < 0.05). Data was presented as number (%) for every group.

The allele A and the genotype AA of rs2278420 in SR group were more common compared to those in CR group (*p* < 0.001 and *p =* 7.61×10^-6^). Under the dominate model, the frequency of rs2278420 genotype AA was higher in SR group than that in CR group, and patients with genotype AA was found to have 6-fold increased risk of developing SR (*p* < 0.001, OR = 6.016, **Table 3**). For rs6509607, the genotype AA and the allele A were more prevalent in SR group compared to those in CR group (*p =* 3.77×10^-4^ and *p* < 0.001). The genotype AA was more likely to be observed in SR group under the dominate model. Likewise, the proportion of genotype GG was significantly higher in CR group under the recessive model (*p* < 0.001 and *p* = 0.015). Moreover, there was 4-fold increased risk of developing SR in patients with genotype AA (OR = 4.138, **Table 3**).

### 3.3 rs2278420 allele G and rs6509607 allele G are associated with HBeAg loss by Peg-IFNα treatment

**Figure 2:**
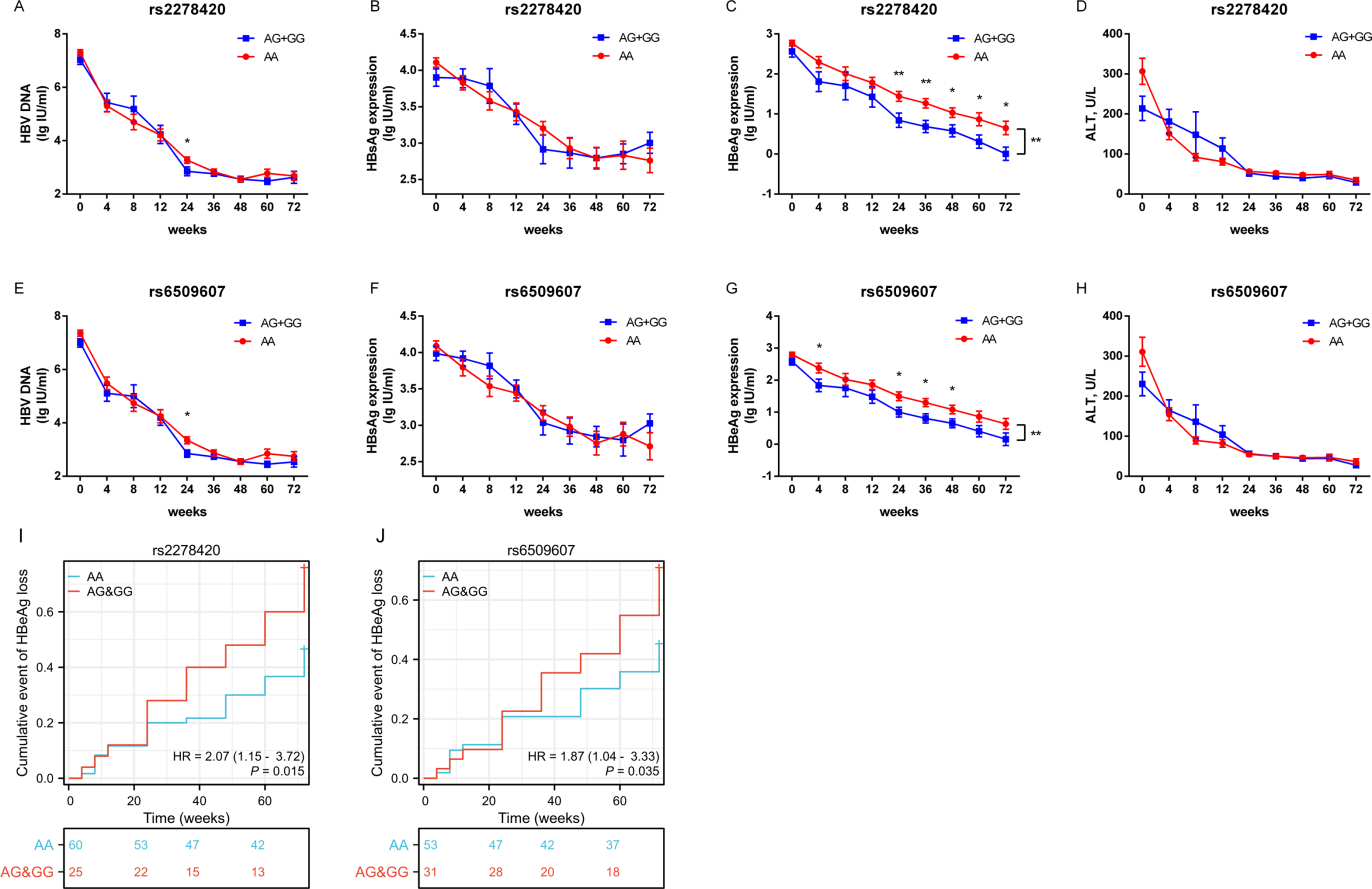
The allele G at both rs2278420 and rs6509607 was associated with HBeAg loss by Peg-IFNα treatment. Serum levels of **(A, E)** HBV DNA, **(B, F)** HBsAg, **(C, G)** HBeAg, and **(D, H)** ALT in rs2278420 (AG+GG) (blue)/AA (red) genotypes and rs6509607 (AG+GG) (blue)/AA (red) genotypes of all CHB patients at 0, 4, 8, 12, 24, 36, 48, 72 weeks since Peg-IFNα treatment. Generalized estimating equations were used to analyze the differences in overall changes of viral indicators between different genotypes, while t-tests were used to analyze the differences at a single time point between different genotypes. **(I, J)** The cumulative event of HBeAg loss was analyzed with cox regression analysis. Error bars, SEM. * *p* < 0.05, ** *p* < 0.01.

The serum levels of HBV DNA, HBsAg, HBeAg and ALT, as well as the rates of HBeAg loss, were observed at specific time points over 72 weeks from the start of Peg-IFNα treatment in all HBeAg-positive CHB patients. The results showed that the levels of HBV DNA, HBsAg, HBeAg, and ALT decreased significantly as the treatment duration increased, but the trends in the decline of HBsAg differed between the two genotypes of both SNPs (all *p <*0.05, **Supplementary Table S2**). The mean concentration of HBeAg in patients with genotypes (AG+GG) was lower than those with genotype AA during Peg-IFNα treatment (*p*_rs2278420_ = 0.004, **Figure 2C** and *p*_rs6509607_ = 0.009, **Figure 2G**, **Supplementary Table S2**). From a temporal perspective, at 24, 36, 48, 60 and 72 weeks of Peg-IFNα treatment in patients with rs2278420 genotype (AG+GG), and at 4, 24, 36 and 48 weeks of Peg-IFNα treatment in patients with rs6509607 genotype (AG+GG), the mean concentration of serum HBeAg were significantly lower than those with genotype AA. (rs2278420: *p*_24W_= 0.009, *p*_36W_ = 0.005, *p*_48W_= 0.021, *p*_60W_= 0.030 and *p*_72W_= 0.019, **Figures 2A–D**; rs6509607: *p*_4W_= 0.031, *p*_24W_= 0.035, *p*_36W_ = 0.012 and *p*_48W_ = 0.032, **Figures 2E–H**).

Furthermore, the cox regression model of HBeAg loss demonstrated that genotypes of rs2278420 and rs6509607 were independent factors to HBeAg loss (*p* = 0.015 and *p* = 0.035, **Figures 2I, J**). Patients with genotype (AG+GG) had respectively 2.07-fold and 1.87-fold increased probability of developing HBeAg loss (HR=2.07 and HR=1.87, **Figures 2I, J**). Which suggested that the allele G at both rs2278420 and rs6509607 might be related to HBeAg loss in CHB patients receiving Peg-IFNα therapy.

### 3.4 Genotypes of rs2278420 and rs6509607 are associated with ZNF350 mRNA expression

**Figure 3:**
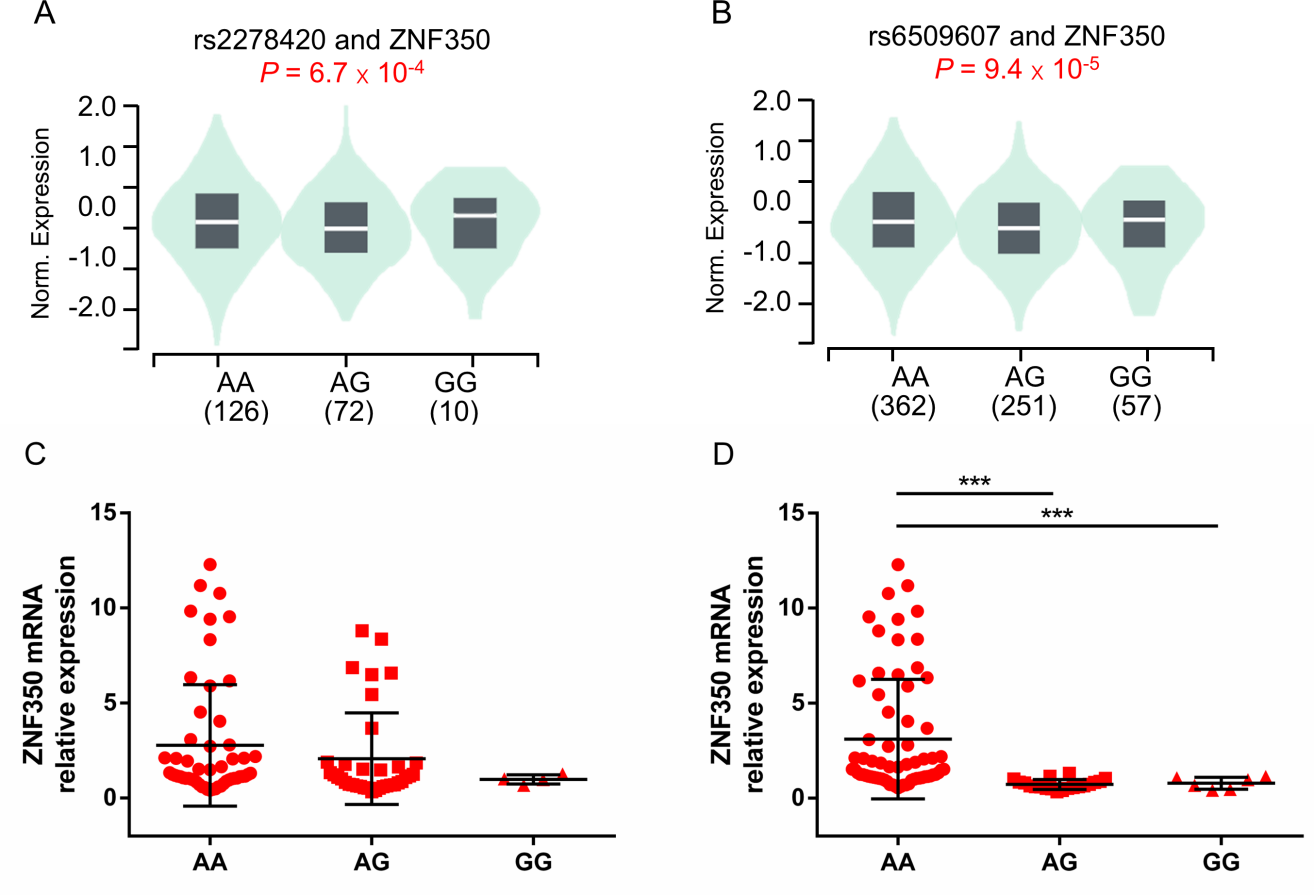
The comparison of ZNF350 mRNA expression with different genotypes of rs2278420 and rs6509607 in the whole blood. Comparison of whole blood ZNF350 mRNA levels in subjects with **(A)** different rs2278420 genotypes (AA, AG and GG) and **(B)** different rs6509607 genotypes (AA, AG and GG), results were shown from healthy samples of GTEx database. The expression of ZNF350 mRNA in AA, AG and GG genotype of **(C)** rs2278420 and **(D)** rs6509607 in HBeAg-positive HBV carriers. *** *p* < 0.001.

To investigate the potential functional genes related to rs2278420 and rs6509607, we conducted eQTL analysis based on GTEx database. The analysis revealed that rs2278420 allele G and rs6509607 allele A were significantly linked to increased ZNF350 mRNA levels, not only in the whole blood (*p* = 6.7×10^-4^ and *p* = 9.4×10^-5^ **Figures 3A, B**), but also in other organs such as liver (*p* = 0.0063 and *p* = 0.18) and thyroid (*p* = 2.2×10^-7^ and *p* = 5.3×10^-7^) according to GTEx data.

To observe whether this correlation between ZNF350 mRNA level and genotype exist in the presence of HBV, we randomly enrolled and genotyped 81 HBV carriers who were HBeAg positive, and detected the mRNA expression of ZNF350. There were 46 patients with AA, 31 patients with AG and 4 patients with GG genotype at rs2278420, while there were 59 patients with AA, 16 patients with AG and 6 patients with GG genotype at rs6509607. The results showed that the level of ZNF350 mRNA was higher in patients with genotype AA than those in patients with genotypes AG and GG at rs6509607 (*p* < 0.001 and *p* < 0.001, **Figure 3D**). Similar results were observed in rs2278420, but they were not statistically significant, probably due to the small number of patients with GG (*p* > 0.05, **Figure 3C**). The results suggested that the allele A at rs6509607 was associated with increased ZNF350 mRNA expression in individuals with HBV infection.

### 3.5 Lower ZNF350 mRNA expression is associated with CR in CHB patients treated with Peg-IFNα

**Figure 4:**
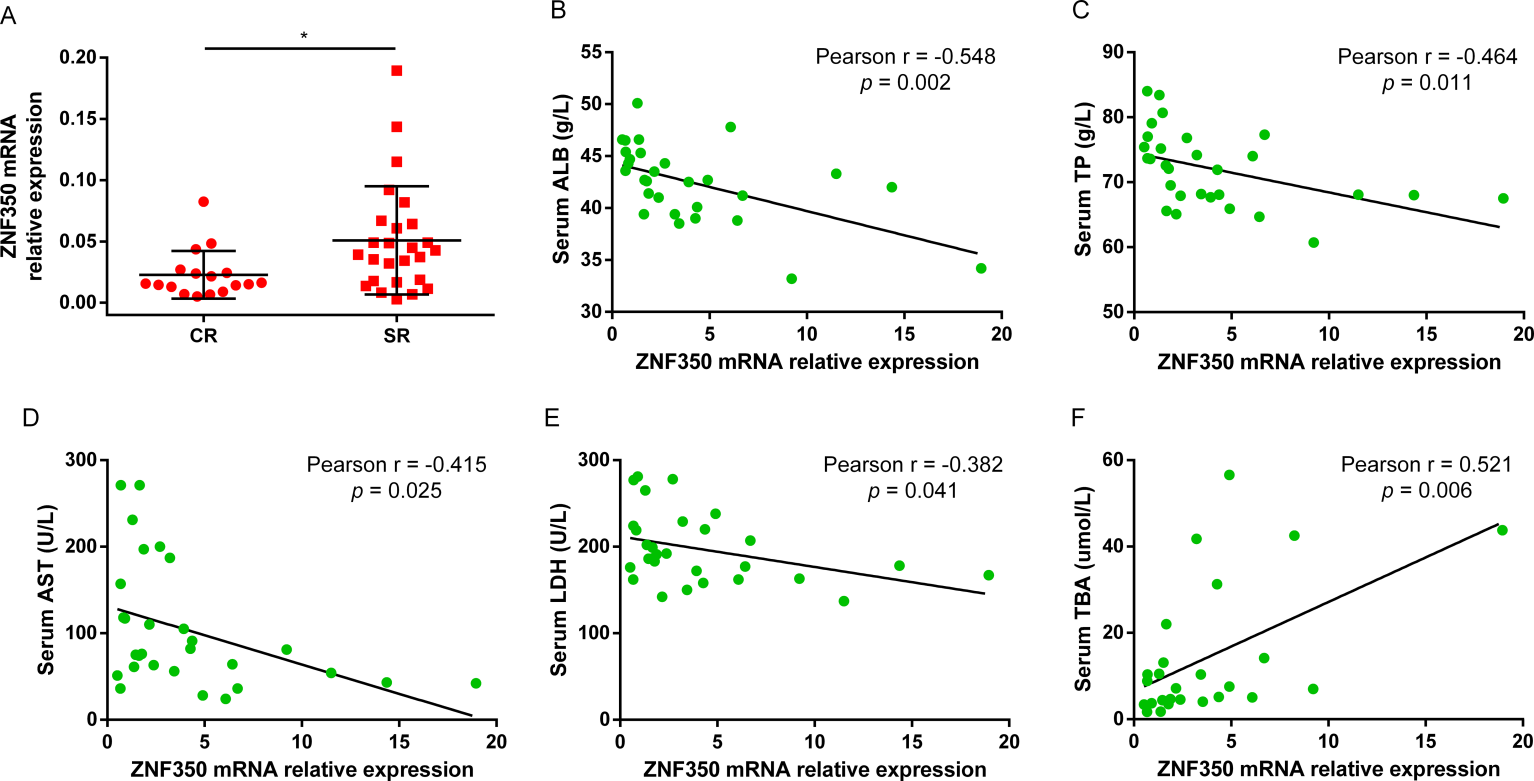
The mRNA level of ZNF350 was associated with CR of Peg-IFNα. **(A)** The mRNA expression of ZNF350 gene between CR group and SR group in CHB patients before Peg-IFNα treatment. **(B-F)** Correlation analysis between the level of ZNF350 mRNA and serum ALB, TP, AST, LDH and TBA. ALB, albumin; TP, total protein; AST, aspartate aminotransferase; LDH, lactic dehydrogenase; TBA, total bile acid. * *p* < 0.05.

To observe whether the expression of ZNF350 gene was associated with the efficacy of Peg-IFNα, the levels of biochemical indicators and ZNF350 mRNA from the whole blood of cohort patients were analyzed before treatment. The results showed that the level of ZNF350 mRNA in SR group was higher than that in CR group (*p* = 0.018, **Figure 4A**), and the correlation analysis demonstrated that the expression of ZNF350 mRNA were negatively correlated with serum levels of ALB, TP, AST, LDH, while positively associated with serum level of TBA (*r*_ALB_ = -0.548, *p* = 0.002; *r*_TP_ = -0.464, *p* = 0.011; *r*_AST_ = -0.415, *p* = 0.025; *r*_LDH_ = -0.328, *p* = 0.041; *r*_TBA_ = 0.521, *p* = 0.006, **Figures 4B–F**).

### 3.6 The genotypes of rs2278420 and rs6509607 are associated with the regulation of IFNα signaling pathway

**Figure 5:**
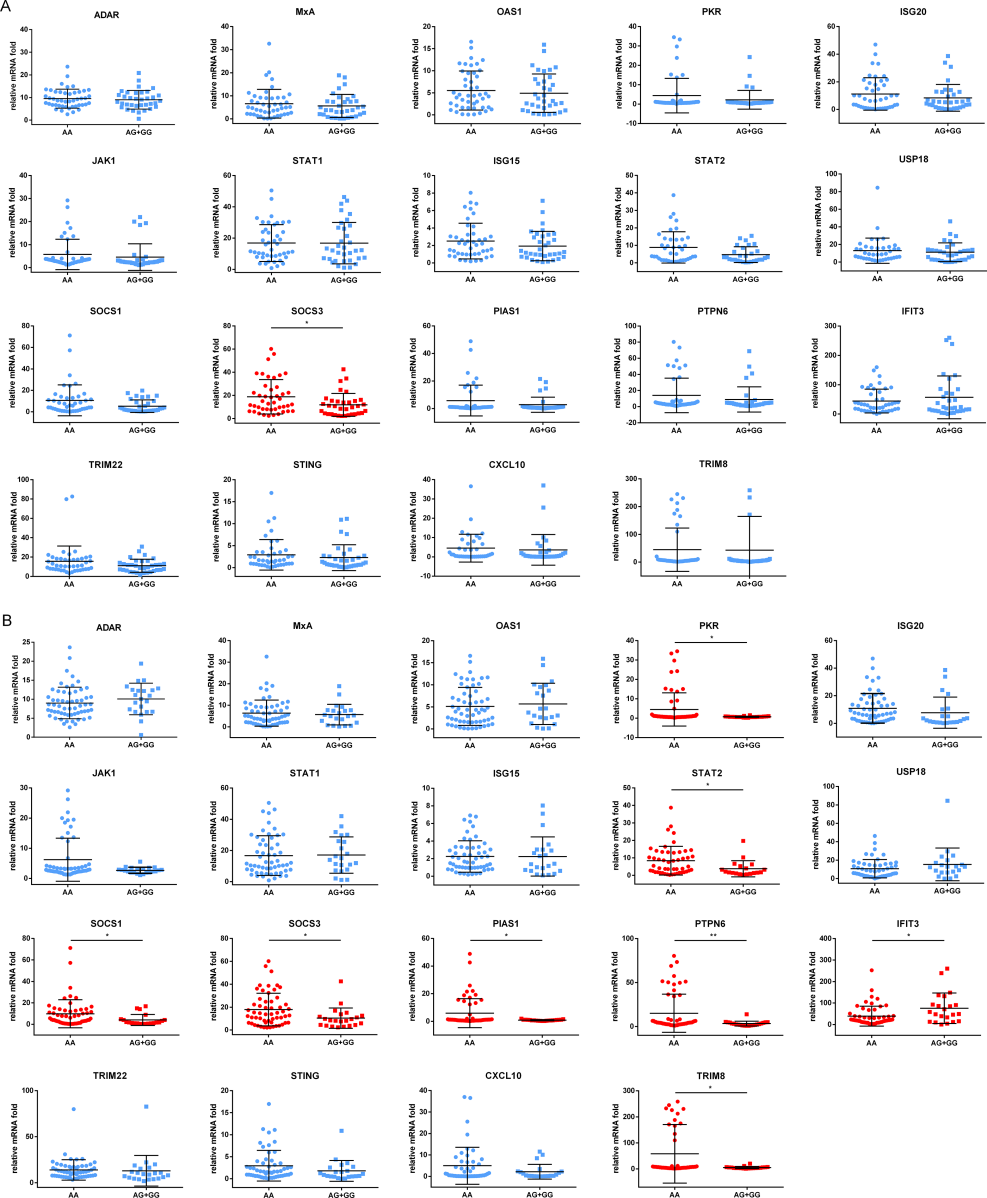
Expression of proteins induced by IFNα in different genotypes of rs2278420 and rs6509607. The peripheral blood mononuclear cells (PBMCs) of patients with **(A)** rs2278420 or **(B)** rs6509607 were cultured *in vitro* and stimulated with IFNα (1000U/L) for 6 hours, then the mRNA levels were detected. ADAR, Adenosine Deaminase; MxA, Myxoma Resistance Protein 1; OAS1, 2’-5’-Oligoadenylate Synthetase; PKR, Double Stranded RNA-activated Protein Kinase; ISG20, Interferon Stimulated Gene 20; JAK1, Janus-activated Kinase Janus 1; STAT1, Signal Transducer and Activator of Transcription1; USP18, Ubiquitin Specific Peptidase 18; SOCS1, Suppressor of Cytokine Signaling1; PIAS1, Protein Inhibitor of Activated Stat 1; PTPN6, Protein Tyrosine Phosphatase, Non-Receptor Type 6; IFIT3, Interferon Induced Protein with Tetratricopeptide Repeats; TRIM22, Tripartite Motif 22; STING, Stimulator of Interferon Genes Protein; CXCL10, C-X-C Motif Chemokine Ligand C-X-C 10. * p < 0.05, ** p < 0.01.

To explore the mechanism by which the antiviral effect of IFNα was affected by genotypes of rs2278420 and rs6509607, freshly isolated PBMCs from 81 patients enrolled above were cultured for 18 hours and subsequently incubated with IFNα for 6 hours. Finally, mRNA was extracted for detection. The mRNA level of SOCS3 gene in patients with rs2278420 genotype AA was higher than those in patients with genotypes (AG+GG) (*p* = 0.027, **Figure 5A**). The mRNA levels of PKR, STAT2, SOCS1, SOCS3, PIAS1, PTPN6, and TRIM8 genes in patients with rs6509607 genotype AA were higher than those in patients with genotypes (AG+GG). In contrast, the mRNA level of IFIT3 in patients with AA was lower than those in patients with (AG+GG) (*p*_PKR_ = 0.029, *p*_STAT2_ = 0.013, *p*_SOCS1_ = 0.030, *p*_SOCS3_ = 0.030, *p*_PIAS1_ = 0.025, *p*_PTPN6_ = 0.003, *p*_TRIM8_ = 0.011, and *p*_IFIT3_ = 0.016, **Figure 5B**). Collectively, these results suggested that the IFNα signaling pathway might be modulated by the polymorphisms of rs2278420 and rs6509607, with the AA genotype likely playing a major role in negative regulation.

## 4 Discussion

To explain individual differences in susceptibility to HBV, disease development, clinical outcomes, and therapeutic efficacy, recent genetic studies have primarily focused on variants of HLA genes, Toll-like receptor pathway-related genes, cytokine genes, and interferon stimulated genes (ISGs) (13, 14). However, limited research has explored the correlation between single nucleotide polymorphisms (SNPs) and the effectiveness of antiviral therapy, with existing studies producing conflicting results (9, 15–17). The poor tolerance and limited efficacy of IFNα treatment necessitate the prediction of responsive patients prior to initiating therapy.

We found for the first time that ZNF350 gene polymorphisms were associated with the efficacy of Peg-IFNα in patients with HBeAg-positive CHB. ZNF350 belongs to Krüppel-associated box domain zinc finger proteins (KRAB-ZFPs) family, which is one of the largest families of transcriptional regulators in higher vertebrates. KRAB-ZFPs act as transcriptional repressors in repression of transposable elements (TEs) and as mediators of DNA methylation (18–20). ZNF350 interacts with a specific consensus sequence within intron 3 of the growth arrest and DNA damage inducible (GADD45) gene, which is induced by BRCA-1 and functioned as a transcriptional repressor (21, 22).

In this study, the lower level of ZNF350 mRNA was associated with complete response of Peg-IFNα in patients with HBeAg-positive CHB, along with higher serum level of ALB and lower serum level of TBA. The stability of the HBV covalently closed circular DNA (cccDNA) in non-dividing hepatocytes is a crucial factor to HBV persistence and limits the effectiveness of antiviral drugs. This residual of cccDNA can be broken down by hepatocyte regeneration, and elevated ALB level can serve as an indicator for monitoring hepatocyte regeneration (23–25). Conversely, abnormal accumulation of bile acids promoted HBV replication and inhibited Peg-IFNα response through impairing the functions of CD8^+^ T cells and NK cells (26). This correlation between ZNF350 mRNA and biochemical indicators such as ALB and TBA reinforced the finding that ZNF350 was a negative factor of Peg-IFNα response.

Our results based on the eQTL analysis revealed that genotypes of rs2278420 and rs6509607 were associated with ZNF350 mRNA level in the whole blood and several other tissues. The expression of ZNF350 might be organ-specific. In the whole blood, the allele G of rs2278420 was reported to be associated with higher ZNF350 mRNA expression, while in the artery-tibial, the allele A was reported to be associated with higher ZNF350 mRNA expression. We observed the same pattern in the artery-tibial in this study. This may be due to the non-disease populations of GTEx data, insufficient sample size, or different physiological states accompanying HBV infection.

The allele G of rs2278420 was reported to significantly influence individual susceptibility to breast cancer (27). In this study, the G allele in both rs2278420 and rs6509607 were found to be associated with a complete response to Peg-IFNα treatment in HBeAg-positive CHB patients, and related to a higher rate of HBeAg loss during Peg-IFNα therapy.

In exploring the mechanism by which gene polymorphisms were involved in the antiviral effect of IFNα, we found that many genes exhibited differential expression across different genotypes of rs6509607. This may be attributed to the variant expression of ZNF350 caused by different genotypes, as ZNF350 may be involved in the regulation of these genes' expression. Similarly, the rs2278420 genotype was related to SOCS3 mRNA level, possibly because the genotype also affected the properties of ZNF350. Although there was interferon stimulated gene (such as TRIM8) expressed higher in the AA genotype of rs6509607, it was primarily the negative regulatory genes of IFNα signaling pathway (such as SOCS1, SOCS3 and PIAS1) that were elevated in the AA genotype. In addition, at both SNPs of the AA genotype was the mRNA level of SOCS3 gene elevated, further indicating that the AA genotype may play a primary role in the negative regulation of IFNα signaling pathway. This also explained why patients with AA genotype of both SNPs had a lower rate of HBeAg loss.

rs2278420 is located not only on the ZNF350 gene, but also on ZNF350-AS1, and its linkage disequilibrium site, rs6509607, is also located on ZNF350-AS1. ZNF350-AS1, known as hepatocellular carcinoma associated transcript 3 (HCCAT3), is a lncRNA which potentially facilitating tumor growth in patients with HBV-related HCC (28). Systematic large-scale studies have shown that abnormal expression of antisense RNA (asRNA) is associated with tumorigenesis (29). AsRNAs could be powerful tools in antiviral and anticancer treatments (30). As mentioned above, asRNA can inhibit the transcription of target mRNA, but whether gene polymorphisms in asRNA affect its inhibitory activity remains unknown. In this study, we also explored the association between ZNF350-AS1 polymorphisms and its expression, but no statistical difference was found. Moreover, eQTL analysis did not show a correlation between ZNF350-AS1 expression and these two SNPs in peripheral blood or liver, but rs6509607 was found to have a strong correlation with ZNF350-AS1 expression in the testis and thyroid, so no further study was conducted.

In conclusion, our study reveals that polymorphisms in ZNF350 gene are associated with the efficacy of Peg-IFNα in patients with HBeAg-positive CHB, but it has not yet been fully explained how SNPs regulate IFNα signaling pathway. In the future, lager multicentric studies are needed and the mechanisms by which gene polymorphisms influence the efficacy of IFNα still require further investigation.

## Data Availability

The datasets presented in this study can be found in online repositories. The names of the repository/repositories and accession number(s) can be found below: GSE276479 (GEO).
