# Supplementary material for: ZNF350 gene polymorphisms promote the response to Peg-IFNα therapy through JAK-STAT signaling pathway in patients with chronic hepatitis B
Source: Front Immunol. 2024 Nov 7;15:1488055. doi: 10.3389/fimmu.2024.1488055 (PMC11578980; doi:10.3389/fimmu.2024.1488055)
Supplement: Supplementary file 1 [file Table1.docx]

***Supplementary Material***

**Supplementary Table 1** Primers for each gene and SNP

| **Gene** | **Forward Primer** **5^’^-3^’^** | **Reverse Primer 5^’^-3^’^** |
| --- | --- | --- |
| rs2278420 | TCCTTCTTGACATTGCCAG | AAATACTTGAAACCCGAGGAA |
| rs6509607 | TGACTCAGGGATTTGCAT | CGTGATTCACCCACTTCG |
| GAPDH | CCATGAGAAGTATGACAACAGCC | CCTTCCACGATACCAAAGTTG |
| ZNF350 | ACGTTGAGGCCCTTCTTGTG | GTGTGCCCCAAGAAATGGTG |
| ADAR | GCTAGAGGAAGCCAAAGCCA | GAAGGATCTGGCTGAAGGGG |
| STAT1 | ATCAGGCTCAGTCGGGGAATA | TGGTCTCGTGTTCTCTGTTCT |
| STAT2 | TGGAGAGCCAGCAACATGAG | GAGGGTGTCTTCCCTTTGGC |
| MxA | GACCATAGGGGTCTTGACCAA | AGACTTGCTCTTTCTGAAAAGCC |
| JAK1 | TCAGTGTGGCGTCATTCTCC | CAGTGAGCTGGCATCAAGGA |
| USP18 | GTCCATCCTGGCTGAGTCCT | CAACCAGGCCATGAGGGTAG |
| ISG15 | ATCACCCAGAAGATCGGCG | AGGTTCGTCGCATTTGTCCA |
| ISG20 | GGGAGGTGGGCAAGTATCAA | GATGCAACAGCAAAGGGTGG |
| PKR | GCTTTGGGAACACGAAGAAGG | TGCAGTTTTCATCTGCAGTCC |
| TRIM8 | GCCTACCGCCTCTACCACT | ATTCCTTCGGATCTCCACGTC |
| SOCS1 | CCTGAACTCGCACCTCCTAC | AATAAAGCCAGAGACCCTCCC |
| SOCS3 | CCATTCGGGAGTTCCTGGAC | TTGGCTTCTTGTGCTTGTGC |
| IFIT3 | GAACATGCTGACCAAGCAGA | CAGTTGTGTCCACCCTTCCT |
| TRIM22 | GAGGTCAAGATGAGCCCACA | GCAGCTTTTCCTGACATTCC |
| PTPN6 | AAGTGAAGAAGCAGCGGTCA | GTCTGTCCATCGCGAAATGC |
| STING | GTGGCTTGAGGGGAACCCGC | GGCTGGAGTGGGGCATCTTCT |
| CXCL10 | ACTGCCATTCTGATTTGCTGC | ATGCAGGTACAGCGTACAGT |
| PIAS1 | CTTAGCCTACGCTCACTCCC | CAAGGGGGATGATGGCAACT |

**Supplementary Table 2** Changes of viral indicators from PEG-IFNα treated HBeAg positive CHB patients with different genotypes of rs2278420 and rs6509607

| SNPs | Variable | HBV DNA | |  | HBsAg | |  | HBeAg | |  | ALT | |
| --- | --- | --- | --- | --- | --- | --- | --- | --- | --- | --- | --- | --- |
|  |  | Wald χ^2^ | *p* |  | Wald χ^2^ | *p* |  | Wald χ^2^ | *p* |  | Wald χ^2^ | *p* |
| rs2278420 | Time effect | 1736.156 | **<0.001** |  | 96.372 | **<0.001** |  | 584.155 | **<0.001** |  | 191.132 | **<0.001** |
|  | Genotype effect | 0.400 | 0.527 |  | 0.010 | 0.920 |  | 6.111 | **0.013 *** |  | 0.04 | 0.947 |
|  | Interaction effect | 7.039 | 0.532 |  | 17.039 | **0.030** |  | 6.514 | 0.590 |  | 13.391 | 0.099 |
| rs6509607 | Time effect | 1778.385 | **<0.001** |  | 95.309 | **<0.001** |  | 542.907 | **<0.001** |  | 196.587 | **<0.001** |
|  | Genotype effect | 3.353 | 0.067 |  | 0.005 | 0.945 |  | 5.578 | **0.018 *** |  | 0.155 | 0.694 |
|  | Interaction effect | 9.589 | 0.295 |  | 16.911 | **0.031** |  | 4.754 | 0.784 |  | 8.353 | 0.400 |

Generalized estimating equation was used for analysis. * After the interaction effect is removed and corrected by Bonferroni, the *p* _rs2278420_=0.004, *p* _rs6509607_=0.009.
